# The Treatment of Acquired Flat-Foot

**Published:** 1896-03-28

**Authors:** A. H. Tubby

**Affiliations:** Assistant Surgeon to, and in Charge of the Orthopædic Department at the Westminster Hospital, Surgeon to the National Orthopædic Hospital, and Surgeon to Out-patients, Evelina Hospital for Sick Children.


### MEDICAL PROGRESS AND HOSPITAL CLINICS.

[*The Editor will be glad to receive offers of co-operation and contributions from members of the profession. All letters should be addressed to* THE EDITOR, AT THE OFFICE, 428, STRAND, LONDON, W.C.]

### THE TREATMENT OF ACQUIRED FLAT-FOOT.

By A. H. TUBBY, M.S.Lond., F.R.C.S.Eng., Assistant Surgeon to, and in Charge of the Orthopædic Department at the Westminster Hospital, Surgeon to the National Orthopædic Hospital, and Surgeon to Out-patients, Evelina Hospital for Sick Children.

The treatment of flat-foot in many cases must be both general and local.

**General Treatment.**—If anæmia be present, iron should be given for a considerable period. The best forms are probably Blaud's pills and the Mistura Ferri Composita of the British Pharmacopœia. Objections to the latter are its uninviting appearance and nauseous taste. A good mode of giving the carbonate of iron is in the form of "jelloids." Change of air and rest are often urgently called for. When the rheumatic diathesis is present, salicylate of soda in acute and sub-acute cases, and in the chronic, iodide of potassium and tincture of guiaiacum, will be found serviceable. Gonorrheal rheumatism is very intractable. Iodide of potassium and cod-liver oil are said to do good. When gout is associated with flat-foot, citrate of lithia, citrate of potassium, and piperazin, associated with suitable dietetic treatment, will ameliorate the general condition. In rhachitic flatfoot, cod-liver oil, the phosphate of iron, the hypophosphites of calcium, with plenty of fresh milk and pure air, will go far to effect a cure.

The relief of pain is often a pressing necessity. The surest therapeutic measure is rest, entire and absolute. But, unfortunately, patients rarely understand the full value of complete rest, and, indeed, are often unable, from force of circumstances and surroundings, to obtain it entirely. At the best, the rest is only partial. If the feet are acutely sensitive, the application of extract of belladonna and glycerine, or warm opium lotion, or poppy fomentations, gives temporary relief. Rédard suggests the injection of cocain near the painful joint. But surely it is not advisable to habituate the patient to the use of this drug in so chronic an affection as flat-foot.

**Local Treatment.**—The means we have are: Rest; exercises, passive and active; mechanical support; and operative measures, including forcible manipulation. But the exact line of treatment must depend upon the degree, severity, and duration of the deformity. Briefly, it may be said that in the first and second degrees of flat-foot, rest, passive and active exercises, and mechanical supports should all be employed, but each to an amount varying with the special cause at work and the condition of the muscles and ligaments. To illustrate my meaning, I would instance the case of flat-foot of the first and second degree in an anæmic overgrown girl. It is useless to trust to tip-toe exercises as an immediate remedy. The patient is often physically incapable of raising herself on tip-toe, and, if she do so, the strain on the muscles and ligaments is so great as to cause further breaking down of the arch. This class of case requires rest and support as the prime factors in treatment, and when the parts have regained their positions the muscles may be actively exercised, so that the best means may thereby be taken to prevent a return of the trouble.

**Prophylactic Treatment in Children.**—Royal Whitman,[1] in a most interesting article, has pointed out that "The attitude of muscular weakness, as shown in infancy and old age, is with the feet wide apart and the toes widely separated to increase the base of support. On the contrary, the attitude of muscular strength and activity is with the feet well under the body and the toes pointing straight ahead. The walk of muscular weakness and inactivity is the walk of flat-foot; the step is short and inelastic, the toes widely divergent, the weight borne almost entirely on the inside of the heel. The foot resting flat on the ground is lifted by an exaggerated bend of the knee, the final strain falling entirely on its weakest part" (the arch). He goes on to say that "the walk of civilisation is a direct approximation to the walk of weakness"; because, firstly, the ordinary boots are so made that corns and bunions result, and the muscles are at the same time weakened from disuse by the pressure of the tight, unyielding boot; and, secondly, children are often allowed, and even taught, to stand by drill-masters with the feet abducted, and their inner borders separated at an angle of 60 degrees. Thus the best preventive of flat-foot is a proper walk in shoes not too thick, and approximating in the sole "to the shape of the Greek sandal," so that the foot points directly forwards and is not cramped. All games which fully employ the muscles of the feet and legs should be encouraged.

**Treatment of the First and Second Degrees.**—Mr. Golding-Bird pithily remarks: "The principle of all successful treatment in acquired valgus is to relieve the overstrained adductors, that they may again recover, offer due opposition to the abductors (peronæi) as nearly like that they have lost as possible; relieve the pronation or eversion and the sunken arch, by giving elastic support upwards and inwards. Before this can be done all active spasm on the part of the peronæi must have ceased." In static and rhachitic cases of this degree the first essential is absolute rest, and with this may be combined the relief of the abduction. The patient should be told to sit on a comfortable sofa or bed tailor-fashion, *i.e.*, with the legs crossed and the weight of the legs bearing on the outer edge of the foot. By this posture, which is maintained from two to four weeks, the foot rapidly regains its normal appearance, the pain ceases, the arch rises, and the deformity is temporarily relieved. I have now under my care a butcher who is suffering from acquired valgus in the left foot, and unable to lie up on account of his business. He has adopted the plan of walking on a wooden pin leg, with the leg and foot carried at right angles behind.

But rest merely allows the foot to return to its normal state, and gives the exhausted muscles and weakened ligaments breathing time, so to speak. Now, two other methods should be employed in addition, viz., exercises and elastic support to the arch of the foot, the exact proportion of each being dependent on the general and local conditions.

**Exercises.**—Those most to be recommended are the tip-toe movements. Their object is to strengthen the flexors of the toes, especially the long and short flexors of the great toe. At the same time the tibialis posticus is called into vigorous action, and with the extension of the foot the tibialis anticus comes into play. The exercises should be carefully regulated. The patient stands erect with the arms by the side. The feet are placed on the ground somewhat adducted and inverted, the toes being nearer together than the heels, and the knees fully extended. He then raises himself on tip-toe, and immediately resumes the position of rest. It is best to do this in rhythm to the swing of a pendulum, or to the beat of a metronome, which can be set to the required rate. At first two to three minutes' exercise twice a day, followed by complete rest in the tailor-fashion, is all that can be borne, the patient ceasing the exercise before fatigue is felt. But as the muscles acquire strength and vigour, the duration and the number of times a day may be increased. The same idea is carried out by going upstairs, first bringing the toes in contact with the stair and then the whole foot.

Another form of exercise is to place the feet nearly touching, and to invert the sole so as to bear the body-weight on the outer edge, then to resume the original position. This may be done as often as the tip-toe exercise and in place of it.

Passive exercises may be carried out as follows: The nurse takes the foot and performs a combination of extension movements at the ankle with rotation at the medio-tarsal joint, at first inwards, and then outwards. Later the patient is able to perform them for himself.

**Supports.**—In very slight cases it is sufficient, after resting, to have the heel prolonged forwards on the inner side, and the sole thickened at the inner edge after Thomas' plan. In the first and second degrees of flat-foot, it is worthy of notice that the pain is immediately relieved by temporarily wedging up the inner border of the foot with a thin book or a piece of paper suitably folded. In fact, this forms a good means of diagnosis in doubtful cases.

(1) *Bandages.*—In early cases the application of a wedge-shaped pad of cotton wool, the feet then being strapped with diachylon plaster and bandaged, is much resorted to at some hospitals as a means of relief, especially when the patients are too poor to pay for any kinds of pads in the boots. The strapping is put on in figure of eight fashion from within outwards, each figure of eight being made up of a separate piece, and the bandage is applied from within outwards, so that each turn passing beneath the arch and over the dorsum of the foot pulls the arch upwards and draws the outer border downwards. But this plan has many disadvantages. By the strapping and bandage the foot is cramped and confined, and the cotton wool pad shifts, and becomes useless and uncomfortable. In consonance with the idea of elastic traction, a Martin's rubber bandage has been used. This, however, has disadvantages similar to an ordinary bandage, and may further cause eczema from the confinement of that free perspiration so common in flat-foot.

(2) *Pads and Surgical Soles.*—Pads are made of various materials, viz., felt, wool, leather, steel, rubber, or hollow and filled with glycerine—after Mr. Walsham's pattern. Of these the vulcanised rubber are the most useful, and in the end least expensive, since felt, wool, and leather become sodden with perspiration and hard to the foot, while rubber becomes softer and more elastic with wear. To be of any value, the valgus pad must be made a part of the boot. A good combination for the first and second degrees of valgus is Ernst's boot, with the vulcanised valgus pad and concealed spring. The boot is made on the same pattern as that for varus, with the exception that the spring is on the outer side of the boot. In marked cases, a boot with valgus pad and outside support to the knee with valgus T strap is indicated. At first, pads and surgical boots can be worn for half to one hour, and this time should be gradually increased until the apparatus is worn all day. The surgical sole, which resembles a cork sole with a steel-piece shaped to the concavity of the arch is of little or no value. It does not keep its position, and often does more harm than good.

A most excellent contrivance is Whitman's brace, made of steel or aluminium, which is light and comfortable, and does not interfere with the movements of the foot or muscles. Two objections have been urged against this brace: (1) That it prevents the natural movements of the foot. This is not correct in practice, (2). That it causes painful pressure. This objection is disproved also in practice. When the arch of the foot is nearly restored, the brace acts as a most efficient support; it is also mechanically correct.

Personally, I am content to use the boots with the valgus pad and spring in cases of the first degree, the boot with the outside iron, valgus pad, and T strap in the second degree, and latterly I have used the Whitman's brace and find it very efficient.

**Elastic Tension.**—With the idea of applying continuous upward tension to the sunken arch, various forms of elastic supports have been arranged. Barwell's method is the oldest. Walsham and Hughes have a method of their own, which is a combination of elastic tension with a boot and leg-iron for severe degrees of flat-foot, especially where patients are unable to sufficiently carry out exercises, and where their occupation necessitates the continuance of long hours of standing. Mr. Golding-Bird uses a modification of Barwell's apparatus, in which, by an arrangement of an outside leg-iron, and a broad elastic band, with a simplified attachment to the foot, the strapping in Barwell's apparatus is done away with.

To sum up, then, the treatment of the first and second degrees of flat-foot. First, rest, till the pain has disappeared and the arch is rising; then the use of passive exercises if the muscles continue weakly; but when these become stronger, active exercises. When the patient stands, a suitable support to the arch is required.

[1] Trans. Amer. Orth. Assoc., Vol. I., pp. 122-134.

